# Surgical Aortic Valve Replacement Using a Porcine Model: A Low-Cost Simulation for Surgical Trainees

**DOI:** 10.7759/cureus.66637

**Published:** 2024-08-11

**Authors:** Vincent S Alexander, Michael D Ernst, Christa Haran, Andrew Hines, Andrew D Vogel, Maxwell J Jabaay, Tyler J Wallen, Adam Eppler

**Affiliations:** 1 Department of Research, Alabama College of Osteopathic Medicine, Dothan, USA; 2 Department of Surgery, Mayo Clinic, Rochester, USA; 3 Department of Cardiovascular Surgery, Geisinger Commonwealth School of Medicine, Wilkes-Barre, USA; 4 Department of Cardiovascular Surgery, Southeast Health Medical Center, Dothan, USA

**Keywords:** low-cost simulation, cardiothoracic surgery, porcine model, aortic valve replacement, surgical simulation

## Abstract

Simulation experiences are valuable to the training of future successful surgeons. These experiences introduce trainees to operational concepts through hands-on engagement within a low-stress environment to promote skill, information retention, and increased competency for future success in real-life scenarios. The study aimed to develop a low-cost, reproducible surgical simulation for teaching aortic valve replacement using porcine models. This study employed a single-center educational workshop design to provide trainees with a comprehensive wet laboratory experience in surgical aortic valve replacement using a porcine model. The simulation involved step-by-step procedures using porcine hearts in a wet lab environment, emphasizing specific surgical techniques such as suturing, knot tying, and valve replacement. Simulated valves were created using insulation foaming and aluminum wiring. The study was conducted at a southeastern medical school’s wet lab. Thirty-eight preclinical medical students participated. The simulation was designed to provide a comprehensive overview of the steps involved in aortic valve replacement using porcine models. It emphasized the importance of teamwork, fundamental surgical skills, and effective communication within a surgical setting. The low-cost surgical simulation allowed trainees to learn technical skills that could be tailored to their proficiency level. Simulation for cardiothoracic procedures is limited by monetary spending and the availability of adequate materials to create a beneficial learning experience. This low-cost simulation allows resource-limited institutions to provide their students an additional opportunity to practice fundamental surgical principles such as suturing.

## Introduction

Cardiothoracic (CT) surgery is known to be a high-stake and high-risk surgical field, making it challenging for students and residents to gain meaningful experiences. Thus, their exposure is limited, which can hamper interest in the field [1]. Medical simulation is a well-studied field that provides students with opportunities to gain exposure, practice skills, and fail in a low-stake environment that mirrors the practice environment.

Medical education, mainly surgical training, follows an apprenticeship framework. This format promotes the opportunity to learn with the support of experienced physicians and surgeons, but it is dependent on the time the student or resident has with their attending. Surgical simulations allow the learners to work through these critical surgical skills at a self-promoted pace that is not dependent on their mentor, potentially making their learning experience with their mentor more productive [2,3]. Using simulation allows the opportunity to fine-tune procedural skills before implementing them in the operating room.

The value of simulation in the surgical field has been increasingly incorporated into foundational training, from preclinical medical students to residents. Mastering surgical skills within a low-stress environment is crucial for honing skills, promoting information retention, and building competency for real-life scenarios.

The surgical aortic valve replacement (SAVR) wet lab experience was intended to be a reproducible simulation experience exposing students or residents to essential operational concepts in CT surgery. Potential barriers were removed by creating a low-cost and low-technology simulation experience. While focused on CT surgery, this framework could be adapted to any surgical specialty to enable affordable simulated skills practice. What distinguishes this initiative is its adaptability to learners; it can be taught to medical students or residents, thus catering to a broader medical landscape beyond a single institution. This simulation captures the interest of surgical trainees. It presents a practical solution that overcomes conventional barriers, offering students a way to gain hands-on exposure to surgical skills.

## Technical report

This SAVR wet lab workshop was designed as part of a training modality for practicing fundamental surgical skills, such as knot tying and suturing, and exposing participants to the cardiovascular surgical complexities of valve replacement. The primary audience for this workshop was medical students in their clinical years and surgery residents.

The workshop was conducted in a specialized wet lab environment equipped to facilitate porcine heart manipulation. Each workshop component addresses specific aspects of the aortic valve replacement procedure. The participants engage in a progressively complex series of tasks, from a basic understanding of the pig heart anatomy and suturing to the intricate steps of excising the native valve and implanting a prosthetic valve. This stepwise approach ensures that the learners gradually build their skills and confidence, allowing educators to tailor the experience to the participants' training level.

Participants work in pairs, assuming specific roles like a traditional surgical team. These roles include the primary surgeon and first assistant. The workshop setup allows for an immersive learning experience, where participants develop technical skills and learn the importance of teamwork, communication, and role-specific responsibilities in a high-stake surgical environment.

Objectives

1. Demonstrate essential surgical skills, including precise suturing and tissue handling.

2. Develop a working understanding of the heart’s anatomy with an emphasis on the aortic valve and surrounding structures.

3. Train participants in the safe excision of native aortic valves from pig hearts and preparation for prosthetic valve implantation, focusing on meticulous tissue management.

4. Discuss the SAVR procedure and the intricacies of valve placement using the proper technique.

5. Enhance teamwork and communication skills crucial for successful surgical collaboration, focusing on roles, responsibilities, and effective intraoperative communication in a simulated environment.

Inputs

**Figure 1:**
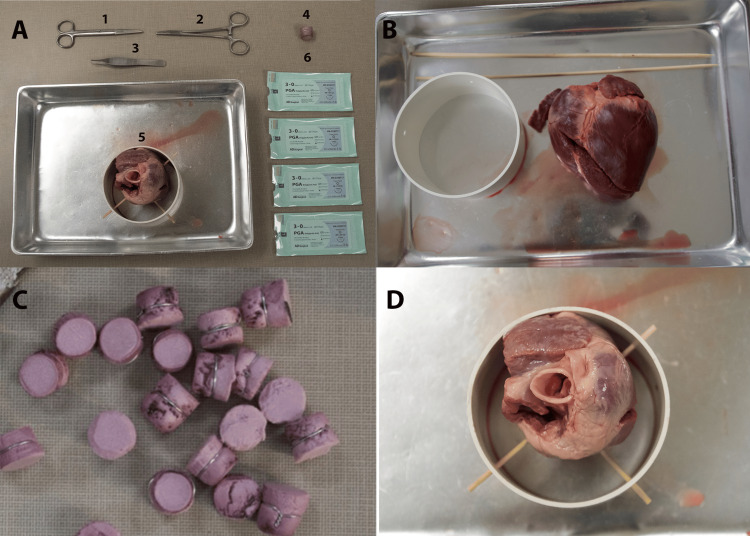
Simulation setup. (A) Displaying of materials used. 1: surgical scissors; 2: needle drivers; 3: Addison tissue forceps; 4: simulated valve; 5: porcine heart positioned inside PVC piping with bamboo skewers; 6: 3-0 sutures. (B) Initial setup before stabilizing the porcine heart with bamboo skewers and PVC piping. (C) Varying sizes of replacement aortic valves are created by insulation foam with aluminum wiring wrapped around it. (D) Porcine heart stabilized in PVC piping with skewers keeping it in place. Special attention should be paid to maximize viewing of the aortic valve leaflets PVC: polyvinyl chloride

Figure 1 shows the simulation setup. Figure 1A lists the props, personnel, and equipment required to run the simulation. Alternatives and substitutions are recommended where applicable. The checklist used for the study is presented in the Appendices.

Wet Laboratory Environment

It is important to ensure the availability of a well-ventilated and controlled setting, like an anatomical laboratory equipped with facilities for proper handling and disposal of organic matter, sharps, and hazardous waste. If a permanent lab is unavailable, consider alternatives like a modular lab unit.

Porcine Hearts

When obtaining the porcine heart, one should emphasize obtaining hearts with minimal damage to the aortic valve and lumen. Hearts could be sourced from local slaughterhouses or through veterinary partnerships. If porcine hearts are unavailable, synthetic heart models (3D soft tissue) should be considered to replicate the aortic valve structure [4].

PVC Piping

PVC piping is used to stabilize the heart model. The piping should have a diameter of 4 inches and a depth of 3 inches, with one hole on each side equally spaced apart. Alternative materials like acrylic or reinforced plastic pipes can also be used.

Bamboo Skewers

Bamboo skewers measure 8 inches long to fit through the holes in the polyvinyl chloride (PVC) piping for additional support. Metal rods or thick wire can be substituted if bamboo is unavailable.

Aluminum Wiring

An 18-gauge aluminum hobby wire emulates the aortic valve. Considering their flexibility and strength, copper or steel wire could be used as alternatives.

Foam Insulation

Rigid foam board insulation sheathing mimics the valve's body. Various sizes should be made to ensure proper fit inside the aortic valve, as there will be variance in size. Alternative materials like silicone or rubber sheets can be used for a more lifelike texture.

Needle Drivers

Needle drivers are essential for suturing. It is important to ensure that a range of sizes is available to accommodate different hand sizes and preferences. Other types of surgical holders may also be used, depending on availability.

Adson Forceps

Adson forceps are ideal for handling delicate tissue. If not available, other fine surgical forceps can be substituted, ensuring a similar level of precision.

Surgical Scissors

Surgical scissors are essential for cutting and dissecting tissue and sutures. Any available scissors can also be used.

Suture Material

The use of a 2-0 suture is recommended. The choice of suture material can vary based on availability and institutional funds. Alternatives like polyglactin or silk sutures can be considered.

Methodology

Lab Preparation

It is necessary to ensure the wet lab is fully prepared two weeks before the simulation. This involves confirming the lab's availability and suitability for the simulation, focusing on adequate ventilation and waste disposal systems. At this stage, coordinate with suppliers to deliver high-quality porcine hearts. For a realistic simulation, these hearts must have well-preserved aortic valves.

Equipment Setup

A week before the simulation, focus on setting up the equipment. This involves assembling the PVC piping framework, which will be used to hold and stabilize the porcine hearts with bamboo skewers (Figure 1B). The foam insulation is prepared by cutting out the simulated valve bodies into various diameters to fit into the lumen of the aortic valve (Figure 1C). Various sizes ensure the ability to let the simulated valve sit well, as there will be variety in lumens for each porcine heart. Then, wrap the aluminum wiring around the body of the foam insulation, ensuring they are correctly sized and ready to mimic the aortic valve structure.

Team Briefing

A detailed briefing session with all participants the day before the simulation is necessary. This briefing is crucial in explaining the objectives, procedures, and especially the safety protocols of the simulation. Clarify each participant's roles and responsibilities, ensuring everyone knows what is expected of them. This is also an opportunity to address any last-minute questions or concerns.

Simulation Day

On the morning of the simulation, it is necessary to conduct a final walkthrough of the lab setup. This ensures that all equipment and materials are correctly placed and ready for use. Brief participants on the specific objectives for the day and make any last-minute adjustments to the setup or roles. Double-check that all safety measures and protocols are in place, ensuring a safe environment for the simulation.

Simulation Execution

The focus is on executing the SAVR procedure steps during the simulation. It is important to closely observe how teams communicate, collaborate, and handle the procedural aspects, and provide guidance or corrective intervention as necessary to ensure the simulation stays on track and educational objectives are met.

Setting up your station

Prepare Your Tools

It is necessary to ensure that scissors, forceps, needle drivers, and several sutures (at least three sets) are organized and that your sutures are 2-0 or 3-0 in size. These are typically used in cardiac procedures for their strength and handling properties, with the option of employing either one-armed or two-armed sutures.

Organize Your Work Area

This includes securing the pig heart in the stabilization device and ensuring good lighting and comfortable access from all sides (Figure 1D).

Leaflet excision

**Figure 2:**
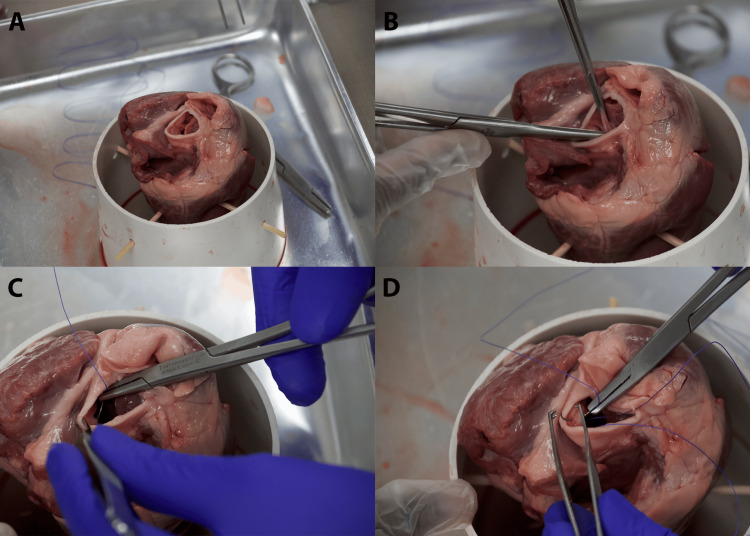
Leaflet excision. (A) Ensuring proper visualization of the aortic leaflets to make sure maximum viewing for precise excision. (B) Utilizing Addison forceps to hold leaflets. Excise the leaflets at the annulus. (C) Needle drivers are utilized to place the suture at the aortic annulus. Practice the supination of the needle driver at this step. (D) Placement of at least three total sutures at the nadir of each cusp; this will create a triangle

Figure 2 shows the leaflet excision.

Visual Inspection

Visual inspection involves identifying the three leaflets of the aortic valve within the heart's anatomy (Figure 2A).

Careful Excision

Then, surgical scissors are used to carefully excise the leaflets, cutting at the base where the leaflets meet the aortic annulus. The aim is to preserve as much of the annular structure as possible to facilitate suture placement (Figure 2B).

Placing Sutures

Figure 2C shows the way sutures are placed in the aortic annulus. Needle drivers are utilized to place the suture at the aortic annulus.

Placement

**Figure 3:**
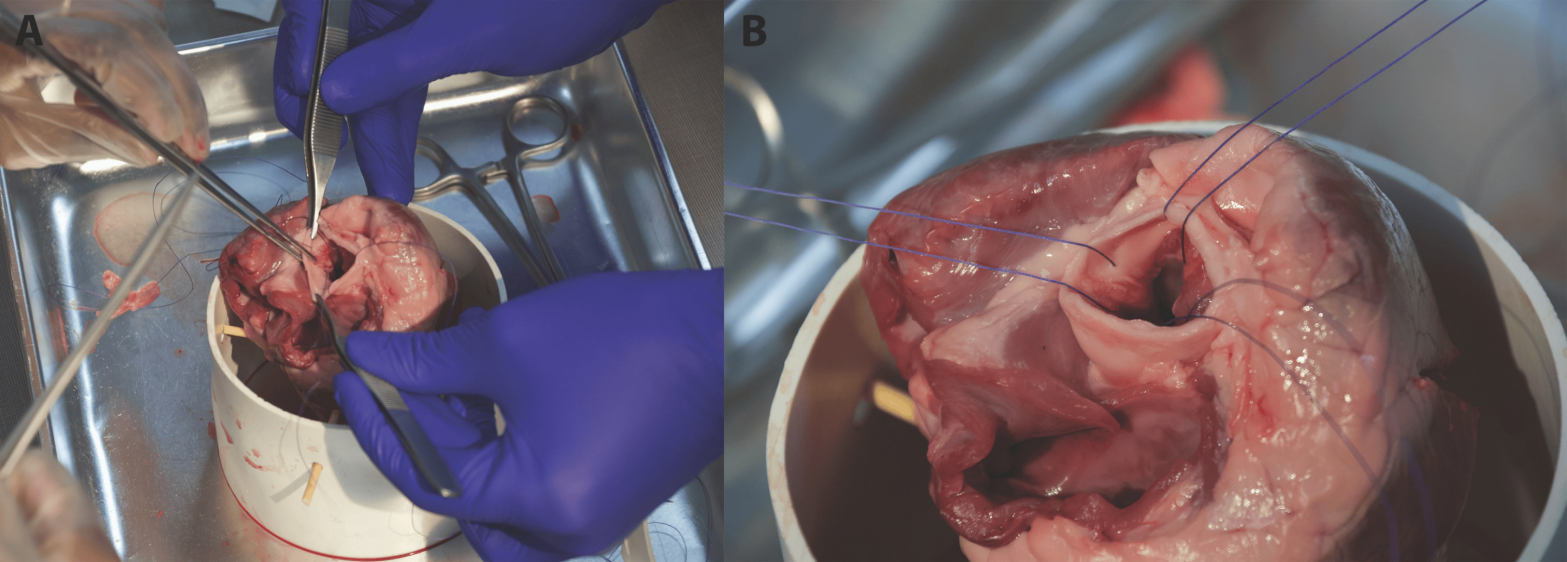
Suture placement. (A) Placement of at least three total sutures at the nadir of each cusp. (B) Ensuring the organization of the suture threads, as they will be used as guides to thread the replacement valve inferiorly into the lumen. This can also be done with a snap or mosquito

Figure 2D shows inserting the needle through the annulus at evenly spaced intervals. Approximately 9-12 sutures are typically placed around the annulus. However, if needed for time management or due to suture availability, this can be reduced to three total sutures (with one placed either at the nadir of each cusp or at the apex of each commissure) (Figures 3A, 3B).

Attaching sutures to the prosthetic valve sewing ring

**Figure 4:**
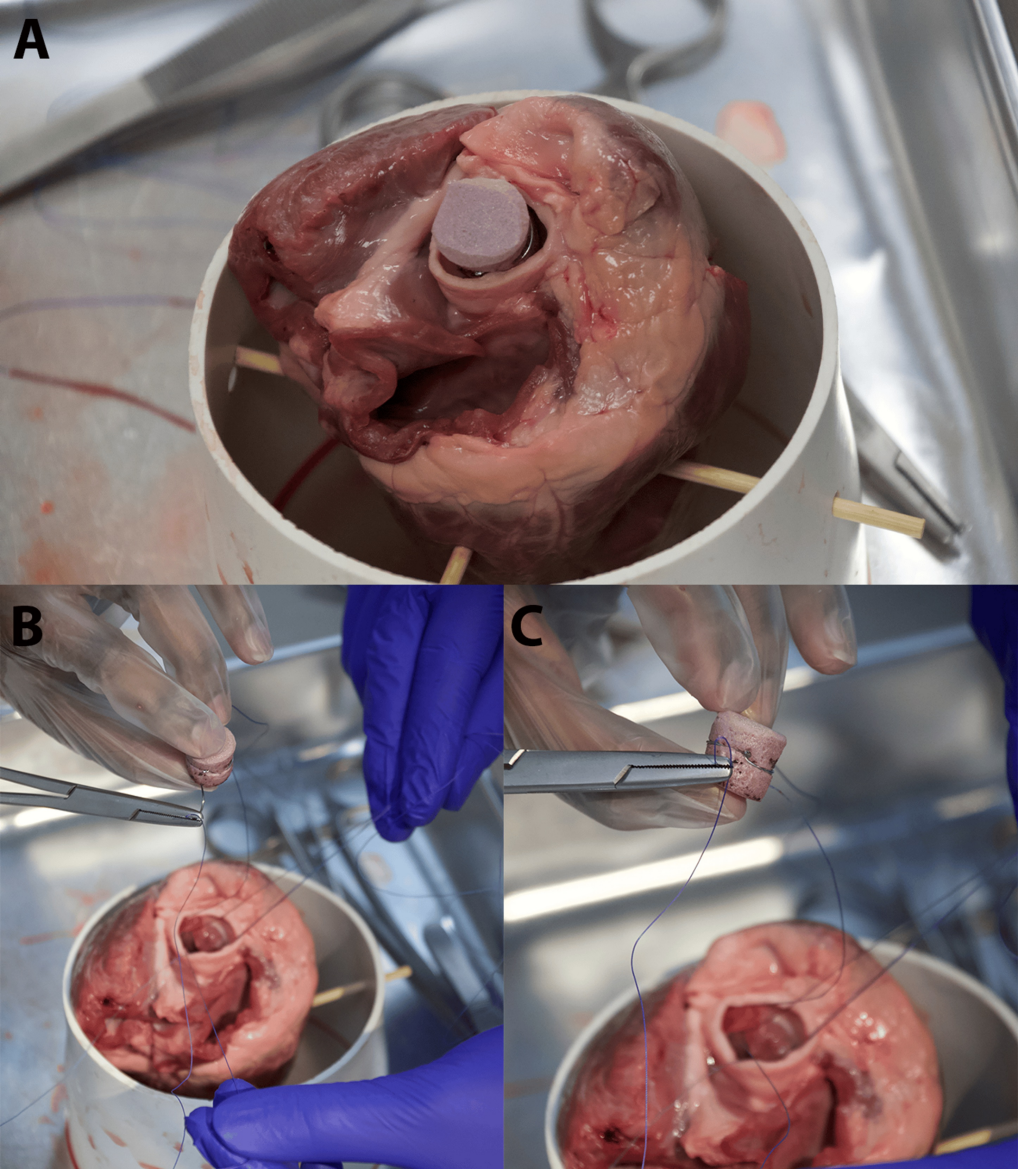
Placing the simulated valve. (A) Ensure the proper sizing of the simulated valve to ensure its proper placement in the aorta's lumen. (B) Place each needle of the double-armed suture through the prosthetic valve's sewing ring (aluminum wire). (C) Place each needle of the double-armed suture through the prosthetic valve's sewing ring (aluminum wire)

Figure 4 shows how the simulated valve is placed.

Aligning the Valve

It is important to ensure that the prosthetic valve is oriented correctly so that the leaflets open toward the aorta, allowing blood to flow from the ventricle to the aorta and is the suitable size (Figure 4A). Also, ensure that the valve size will fit into the lumen of the aorta. If no valve will fit well, prioritize a small valve, as there will be more ease with manipulation.

Securing Sutures

**Figure 5:**
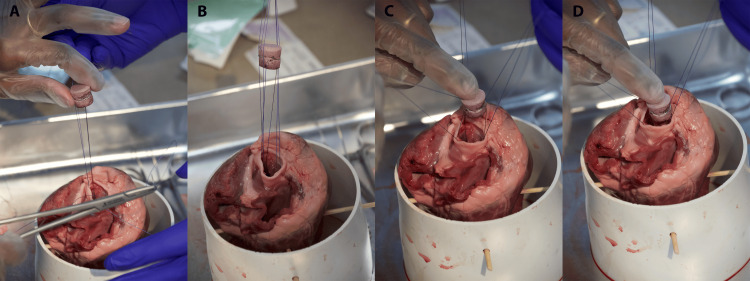
Guiding the simulated valve into proper placement. (A) Ensure appropriate spacing to distribute the tension evenly. Single-armed sutures can also be used if needed. (B) Ensure suture insertion allows for ease of placement into the lumen of the aorta. It should glide easily down. (C) While holding the suture threads, move the replacement valve inferiorly so that it will be seated at the previous aortic leaflet site. (D) Ensure the proper organization of the suture thread with the help of your assistant to avoid entanglement of the suture

Place each needle of the double-armed suture through the prosthetic valve's sewing ring (aluminum wire) (Figure 4B). Ensure appropriate spacing to distribute the tension evenly (Figure 4C). Single-armed sutures can also be used if needed. The assistant should help with suture management, keeping previously placed sutures out of the way (Figures 5A, 5B).

Seating the valve and tying off sutures

Lowering and Positioning the Valve

Once all sutures are placed, cut off each needle and dispose of them in a sharps container. While gently pulling upward on all sutures, carefully lower the valve into position within the aortic annulus, using the sutures to guide it into place (Figures 5C, 5D).

Final Knot Tying

**Figure 6:**
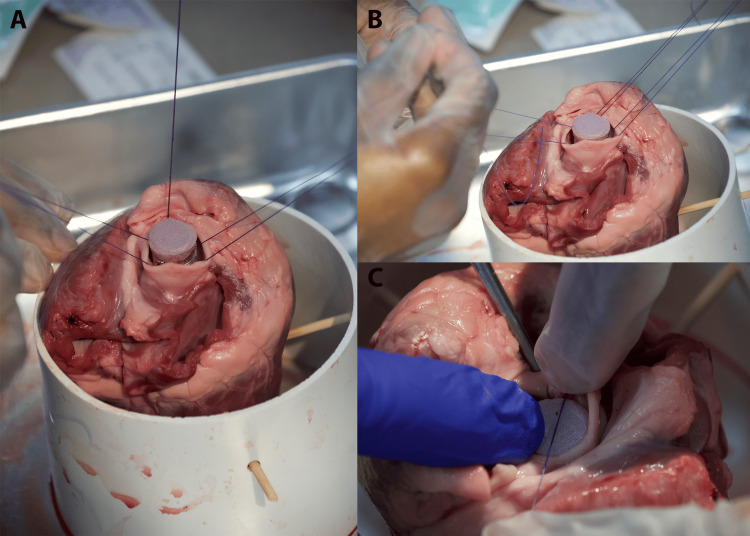
Securing the simulated valve into place. (A) Once the replacement aortic valve is placed at the excised site, prepare for two hand knots to be placed to secure the valve's placement. (B) Have the assistant move the suture threads to the side for the knots to be thrown. (C) Ensure proper placement of the knot inferiorly at the annulus of the aorta

Figure 6 shows how to secure the valve by tying each suture with one or two-handed knots.

Valve assessment and adjustment

Inspect the Valve Placement

**Figure 7:**
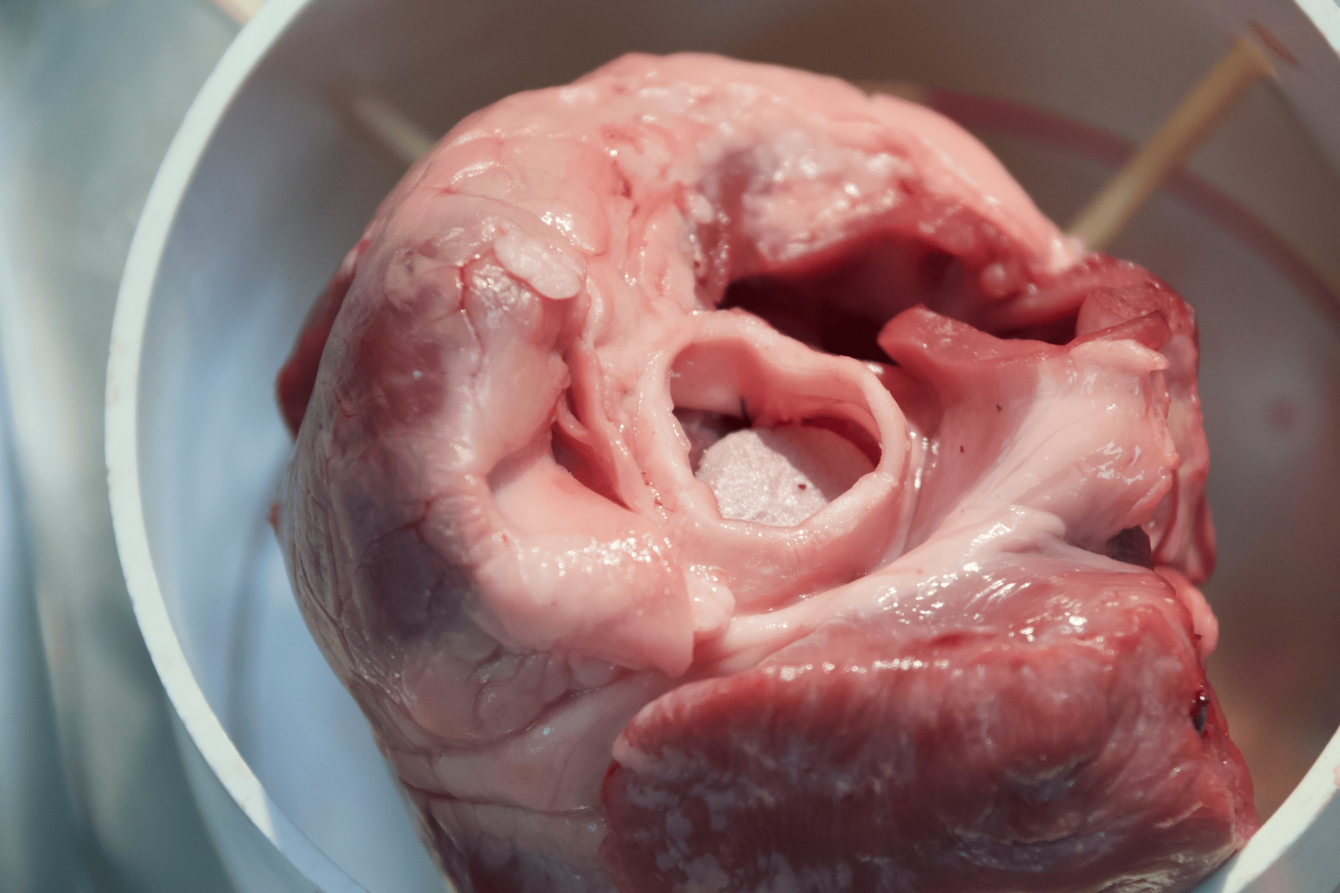
Finalized simulation. Once all knots have been placed and the valve is properly secured, cut away the remaining suture threads with scissors

Verify that the valve is seated correctly and has no visible gaps (there will be gaps if fewer sutures are placed due to limited availability), as shown in Figure 7.

Simulate Blood Flow

If possible, simulate blood flow (using water or another fluid) to check for leaks. Adjust any sutures if necessary to ensure a tight seal around the valve.

Postsimulation review

Gather all participants for a debriefing session after the simulation. Discuss the simulation's outcomes, including what was successful and areas for improvement. This session is crucial for reflective learning, allowing participants to share their insights and learn from each other's experiences.

Cleanup and disposal

Guide participants through the proper cleanup of the lab space, emphasizing the importance of responsible disposal of organic materials and adherence to safety and ethical guidelines. Ensure that all reusable equipment is sterilized and stored appropriately for future use.

Results

The SAVR wet lab workshop was successfully completed in a rural medical school in the Southeast United States. Thirty-eight preclinical medical students completed the simulation under guidance from a board-certified CT surgeon. Additional third- and fourth-year medical students were available to provide immediate assistance to participants during the simulation. These students had previously completed the simulation and knew the appropriate steps. The workshop was designed to provide a comprehensive overview of the steps involved in aortic valve replacement using porcine models. It emphasized the importance of teamwork, fundamental surgical skills, and effective communication within a surgical setting. In April 2024, the total cost per model was $2.02, with donated hearts from a local butchery. A local pig butcher was located in the periphery of the rural medical school. However, it costs $9.01 per student if purchasing porcine hearts is needed, with readily available porcine hearts online.

Challenges such as variability in the anatomical quality of porcine hearts were noted. The workshop addressed these challenges through adaptive learning strategies, including enhanced visual aids and targeted instructor interventions, ensuring that learning objectives were met despite these obstacles.

## Discussion

Surgical simulation is an emerging field with many surgical subspecialties beginning to implement more simulation experiences for trainees. These experiences are integral to shaping the skills of future surgeons by providing a nuanced and hands-on approach to familiarizing trainees with essential operational techniques [5,6]. Surgical simulation trainees gain exposure to surgical techniques in low-stake environments while allowing students to explore potential interest in surgery. While there is an increasing focus in the literature on the benefits of surgical simulation in improving operational competency, there is still much to be learned regarding the use of low-cost surgical simulation experiences as a method of helping medical students explore potential interest in surgery. A significant challenge in surgical simulation lies in the limited resources and availability of simulators, particularly in institutions without a surgical residency program.

Surgical simulation is not unique to CT surgery. General surgery and several surgical subspecialties, including neurosurgery, otolaryngology, ophthalmology, and urology, have recognized the utility of simulation and have been implementing surgical simulation experiences in their training models [7-12]. A study that involved a bibliometric analysis of 5,285 articles regarding surgical simulation identified laparoscopic skill, three-dimensional printing, and virtual reality as the top three modalities for surgical simulation [13]. Advanced technologies such as three-dimensional printing are currently used in most surgical subspecialties, such as neurosurgery, to create high-fidelity simulation models [14]. While these simulation modalities appear to be effective, they require resources that most rural institutions do not have, thus limiting the ability of students at those institutions to gain exposure to these types of hands-on surgical simulation experiences.

Specific simulations to learn individual procedures are becoming more accessible for trainees without expecting a significant financial burden. A review article described how 16 different microneurosurgical cranial approaches were simulated using multiple nonliving animal models such as sheep, cows, and swine. These neurosurgery wet laboratory experiences provided cost-effective operational experiences without compromising the intricate nature of the operations [8].

Utilizing animal organs is not a novel development. Bovine surgical simulation is being used in orthopedic surgery. One report described an alternative method for orthopedic surgery residents and attendings using an open reduction, internal fixation standard fixation set, and a bovine or porcine tibia or radius model [15]. These simulation experiences highlight the variety of surgical subspecialties currently embracing simulation and the legitimacy of affordable simulation experiences.

A common theme among surgical specialties about surgical simulation is the emphasis on simulation as an efficacious tool for improving clinical skills. However, there seems to be much less focus in the literature on the use of surgical simulation as a method for allowing students to explore interest in surgery. Much of the literature focuses on medical students or surgical residents acquiring surgical skills [13]. While these are valuable skills to be learned, medical students have little exposure to hands-on surgical techniques to determine potential interest in surgical fields. Medical students have the opportunity to gain hands-on operational experience during clinical rotations. However, the SAVR wet laboratory experience described focuses on a reproducible model whereby students can gain operational exposure earlier in their training. Doing so provides students more opportunities to explore interest in the surgical fields earlier in their training.

The framework utilized to perform this wet laboratory experience could also have implications beyond providing opportunities for operational exposure to medical students. The idea of low-cost surgical simulation under the direction of experts in the field could make it possible for medical professionals in resource-poor countries to create operational opportunities for learning. With the use of video calls, medical professionals may be able to guide learners through wet laboratory experiences like the one described in this paper in areas that may not be reasonable otherwise [16]. This study has limitations, including using porcine models that may not fully mimic human cardiovascular anatomy or surgical conditions. Additionally, the low-cost materials and single-institution participant pool might limit the generalizability and direct applicability of the findings to clinical practice.

## Conclusions

This porcine SAVR wet laboratory experience demonstrates how surgical simulation opportunities can be created with limited resources and help trainees gain a better appreciation for CT surgery. What distinguishes this initiative is its adaptability, catering to a broader medical landscape beyond a single institution, and its ability to scale from the technical perspective of a medical student to a surgical resident. It serves as a pragmatic solution for medical institutions or community hospitals facing financial constraints or limited access to specialized equipment, such as those associated with a heart valve company. This CT surgery wet laboratory experience captures the interest of surgical trainees. It presents a practical solution that overcomes conventional barriers, offering students a unique opportunity to gain exposure to surgical skills in hopes of fostering the next generation of surgeons.

## Figures and Tables

**Figure 8:**
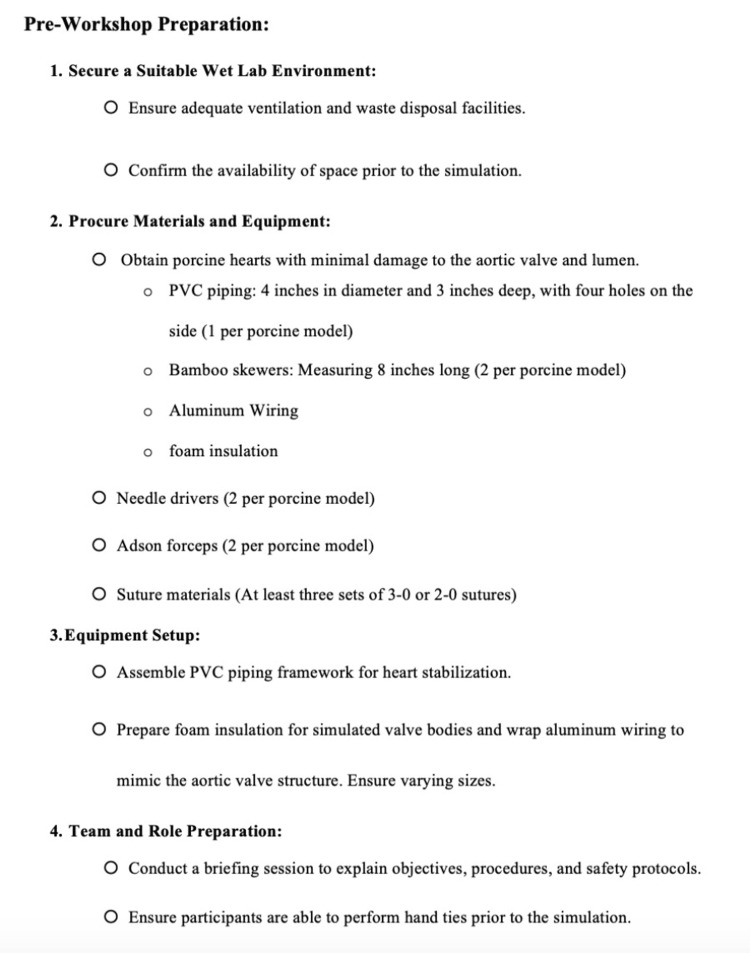
Simulation lab checklist
